# Rapid Bacterial Community Changes during Vermicomposting of Grape Marc Derived from Red Winemaking

**DOI:** 10.3390/microorganisms7100473

**Published:** 2019-10-19

**Authors:** María Gómez Brandón, Manuel Aira, Allison R. Kolbe, Nariane de Andrade, Marcos Pérez-Losada, Jorge Domínguez

**Affiliations:** 1 Grupo de Ecoloxía Animal (GEA), Universidade de Vigo, E-36310 Vigo, Spain; aira@uvigo.es (M.A.); jdguez@uvigo.es (J.D.); 2 Computational Biology Institute, Milken Institute School of Public Health, George Washington University, Ashburn, VA 20147, USA; akolbe@email.gwu.edu (A.R.K.); mlosada323@gmail.com (M.P.-L.); 3 Departamento de Solos, Universidade Federal de Santa María, CEP: 97105-900 Santa María, Río Grande do Sul, Brasil; narianedeandrade@hotmail.com; 4 CIBIO-InBIO, Centro de Investigação em Biodiversidade e Recursos Genéticos, Universidade do Porto, Campus Agrário de Vairão, 4485-661 Vairão, Portugal; 5 Department of Biostatistics and Bioinformatics, Milken Institute School of Public Health, George Washington University, Washington, DC 20052, USA

**Keywords:** winery wastes, earthworms, microbial communities, bacterial succession, core microbiome, vermicompost

## Abstract

Previous studies dealing with changes in microbial communities during vermicomposting were mostly performed at lab-scale conditions and by using low-throughput techniques. Therefore, we sought to characterize the bacterial succession during the vermicomposting of grape marc over a period of 91 days in a pilot-scale vermireactor. Samples were taken at the initiation of vermicomposting, and days 14, 28, 42, and 91, representing both active and mature stages of vermicomposting. By using 16S rRNA high-throughput sequencing, significant changes in the bacterial community composition of grape marc were found after 14 days and throughout the process (*p* < 0.0001). There was also an increase in bacterial diversity, both taxonomic and phylogenetic, from day 14 until the end of the trial. We found the main core microbiome comprised of twelve bacterial taxa (~16.25% of the total sequences) known to be capable of nitrogen fixation and to confer plant-disease suppression. Accordingly, functional diversity included increases in specific genes related to nitrogen fixation and synthesis of plant hormones (salicylic acid) after 91 days. Together, the findings support the use of grape marc vermicompost for sustainable practices in the wine industry by disposing of this high-volume winery by-product and capturing its value to improve soil fertility.

## 1. Introduction

Vermicomposting systems sustain a complex food web, in which the joint action of earthworms and microorganisms ultimately accelerates the stabilization of organic matter [1]. The direct impact of earthworms on microbial communities takes place during transit through the earthworm gut [2], where some microorganisms are digested, while others survive or flourish [3,4]. The changes made through gut associated processes (GAPs) may influence the decomposition process because microorganisms are released again into the environment as part of the earthworm casts [5]. In addition, the earthworm-worked material undergoes natural ageing processes (known as cast associated processes, CAPs), which are generally characterized by a continuous decline of labile C and N nutrient pools that sometimes result in a decrease in microbial biomass [6]. Overall, the impact of earthworms on the vermicomposting process can be split into two phases known as active and maturation-like phases [1].

The temporal changes in decaying organic matter and microbial community composition through both gut and cast associated processes during vermicomposting can be understood as an example of heterotrophic ecological succession [7,8]. Such changes will occur gradually as microbial succession progresses and will be inextricably linked to the quantity and quality of available nutrient supplies, as recently reported by Aira et al. [9] regarding the microbiome composition of ageing casts. Although several studies have employed techniques such as denaturing gradient gel electrophoresis, microarrays, and 454-pyrosequencing to target the bacterial communities associated with vermicomposting processes [10,11,12,13,14,15,16,17], most of these previous works have focused on the end product. Nonetheless, an in-depth characterization of the temporal changes in bacterial communities throughout the process may shed light on the underlying biological mechanisms involved in vermicomposting, and in turn onto the properties of the final vermicompost for its further use as plant growth promoter and in plant disease suppressiveness [18].

Nowadays, wineries are considered one of the major agro-industrial sectors around the world [19]. Vermicomposting has been shown to effectively convert grape marc, the major solid by-product derived from wine production to a value-added product characterized by an increased concentration of macro- and micronutrients and reduced phytotoxicity [20,21,22,23,24,25]. Moreover, vermicomposting was shown to modify bacterial communities of grape marc [25,26], but these two previous studies used a lower resolution technique from a taxonomic viewpoint (based on phospholipid fatty acid profiles), and Gómez-Brandón et al. [26] focused on the changes after one single time point (15 days) at lab-scale conditions.

Therefore, to fulfil this knowledge gap, the present study aims to evaluate the changes in bacterial communities in a pilot-scale vermireactor designed to handle large amounts of grape marc over a period of 91 days as previously done by Kolbe et al. [27]. We chose marc obtained through the red winemaking process of the grape variety Mencía due to its great agricultural and economic significance, as Mencía represents 95% of the annual red grape harvest in northwestern Spain. Towards this goal, we coupled 16S rRNA high-throughput sequencing and metataxonomic analysis to characterize the taxonomic and functional diversity of bacterial communities by capturing several stages of the active and the maturation phases of the vermicomposting process.

## 2. Materials and Methods

### 2.1. Grape Marc and Earthworm Species

Grape marc derived from the red winemaking process of the grape variety Mencía (*Vitis vinifera* sp.) was kindly provided by the Abadía da Cova winery located in Lugo (Galicia, NW Spain) and stored at 4 °C until use.

The Mencía grape marc used in this study was turned and moistened with water for two days prior to the trial in order to achieve a suitable level of moisture for the earthworms (85%) [28]. Individuals of the earthworm species *Eisenia andrei* were used in the vermicomposting trial and obtained from a stock culture reared in the greenhouse of our research group.

### 2.2. Vermicomposting Set-up and Sampling Design

Vermicomposting of the Mencía grape marc was carried out in a rectangular metal pilot-scale vermireactor (4 m long × 1.5 m wide × 1 m high) housed in a greenhouse with no temperature control. Further details about the vermireactor set-up and the sampling procedure are given in [27,29]. Briefly, the vermireactor contained a base layer of vermicompost (12 cm height) as a bed for the earthworms prior to adding the grape marc. The initial earthworm population density in the vermireactor was 273 ± 28 individuals m^−2^, including 14 ± 8 mature earthworms m^−2^, 189 ± 7 juveniles m^−2^ and 70 ± 10 cocoons m^−2^, representing a mean biomass of 62 ± 9 g live weight m^−2^. The fresh grape marc (158 kg fresh weight) was added to the bed in a 12 cm layer placed on top of a plastic mesh (5 mm mesh size) into the vermireactor and covered with a shade cloth to keep the moisture level of the grape marc at approximately 85% throughout the trial. No more grape marc was added to the vermireactor after the start of the experiment. The plastic mesh allows earthworm migration and facilitates sampling, while also preventing mixing of the processed grape marc and the vermicompost bedding.

The density and the biomass of the earthworm population were determined periodically by collecting 10 samples (five from above and five from below the plastic mesh) of the material in the vermireactor during the trial (91 days) by using a core sampler (7.5 cm diameter and 12 cm height). For the characterization of the molecular and the microbial properties, the grape marc layer was divided into 5 equal sections, and five samples (10 g) were taken at random from each section at the beginning of the experiment (day 0) and after 14, 28, 42, and 91 days of vermicomposting. The five samples from each section were bulked and stored in plastic bags at −80 °C until analysis.

### 2.3. Microbial Activity

Microbial activity was determined by measuring the oxygen consumption using a WTW OxiTop^®^ Control System (Weilheim, Germany) according to ISO 16072 [30]. This system was applied to solid samples (5 g, fresh weight) at normal moisture without the addition of easily oxidizable substrates.

### 2.4. DNA Sequencing and Bioinformatic Analyses

DNA extraction was performed on 0.25 g (fresh weight) of grape marc using the MO-BIO PowerSoil^®^ kit following the manufacturer’s protocols. DNA quality and quantity were determined using BioTek’s Take3™ Multi-Volume Plate as previously described [27]. We amplified and sequenced a fragment ~250 bp long of the 16S rRNA gene covering the V4 region by using the primer set 16Sf/16Sr with a dual-index sequencing strategy as described by Kozich et al. [31]. A total of 25 DNA samples representing the different time points (0, 14, 28, 42, and 91 days) were sequenced using the Illumina MiSeq platform at the Genomics Core Facility of the Universitat Pompeu Fabra (Barcelona, Spain). One sample from the initial grape marc did not amplify and was not included in the analysis.

DADA2 (version 1.9) was used to infer the amplicon sequence variants (ASVs) present in each sample [32]. Filtering was performed using standard parameters, with forward reads truncated at 200 nt and reverse reads at 120 nt, and a maximum of two expected errors per read. Default settings were used for ASV inference and chimera detection. The taxonomic assignment was performed against the Silva v132 database using the *assignTaxonomy* function in dada2, which implements RDP naive Bayesian classifier [33,34]. The minimum bootstrap confidence for assigning taxonomy was 80. A total of 2,171,729 sequences (mean: 90,488, SD: 29,411) passed all quality filters and were assigned to 7,163 ASVs.

Putative functional genes involved in nitrogen fixation and synthesis of salicylic acid, which can be considered as a proxy for plant growth and development, were predicted using the Phylogenetic Investigation of Communities by Reconstruction of Unobserved States software package (PICRUSt) [35], which was designed to computationally infer metagenome functional contents from 16S rRNA gene sequences. It is worth noting that our validations (of PICRUSt) on non-human associated environments indicate that the overall predictions perform better than random, but nonetheless we expect that many niche-specific functions will be poorly represented. Briefly, we first picked closed referenced operational taxonomic units (OTUs) (at 97% identity) against the 13_5 version of Greengenes database. The resulting OTU table was then normalized to account for known/predicted 16S copy number over which and the functional composition of our metagenomes was predicted. The weighted nearest sequenced taxon index (NSTI) for our samples was 0.08 ± 0.02 (mean ± s.d.), which indicates that PICRUSt is expected to produce reliable results [35]. Predicted metagenomes were collapsed using the Kyoto Encyclopedia of Genes and Genomes (KEGG) Pathway metadata [36].

Sequence data have been uploaded to the Sequence Read Archive database under accession number PRJNA555188.

### 2.5. Statistical Analysis

We filtered the data set removing ASVs with less than three sequences and not present in at least 5% of samples. By doing this, we removed 77% of ASVs but only 3% of sequences. Rarefaction curves indicated that the sampling depth was optimal for all samples in the full data set (7163 ASVs and 2,171,729 sequences, Figure S1) and the filtered data set (1646 ASVs and 2,106,626 sequences, Figure S2). We normalized ASV counts using the variance-stabilizing transformation for analysis that assumes homoscedasticity or could be influenced by unequal variances [37]. We used raw ASV counts when analysing differential ASV abundances with negative binomial models as implemented in the package DESeq2 [37,38]. Differential abundances of ASVs and other bacterial taxa (phylum and class) were determined according to Wald tests and p-values adjusted by false discovery rate (FDR < 0.05). Since multiple pairwise Wald tests were conducted for each time to time comparison (0–14, 14–28, 28–42, and 42–91 days), we further adjusted these p-values using the Benjamini–Hochberg method to correct for these multiple pairwise comparisons. After correction, non-significant contrasts were considered to have an effect size (log2 fold change) of zero.

An approximately maximum-likelihood phylogenetic tree was inferred using FastTree 2.1 [39]. We defined the core microbiome of vermicomposting of the grape marc as that comprised of ASVs present in all the samples processed by earthworms, that is, samples of 14, 28, 42, and 91 days. Taxonomic α-diversity was calculated as the number of observed ASVs, and diversity and richness were estimated with Shannon and Chao1 indexes, respectively. Phylogenetic diversity was calculated as Faith’s phylogenetic diversity [40]. Taxonomic β-diversity at the ASV level was estimated as the difference in the composition of the bacterial taxonomic community between samples from different times during vermicomposting. This was done by coupling principal coordinate analysis (PCoA) with distance matrices that take the abundance of ASVs into account (Bray–Curtis) or not (Jaccard). Phylogenetic β-diversity was also estimated by PCoA of weighted (considering abundance of ASVs) and unweighted unifrac matrix distances [41] by using the phyloseq library [37]. Mixed models were applied using the ‘nlme’ R package [42] to evaluate the effect of time on α- and β-diversity (PCoA scores) of the grape marc bacterial communities. Time was the fixed factor, while repeated measures were accounted for by considering the effect of time nested in each sample as a random factor. The normality of residuals and homogeneity of variance across groups was checked for each variable. Tukey’s test was used for post-hoc comparisons, and Benjamini–Hochberg FDR was used as a multiple test correction method using the ‘multcomp’ package in R [43].

Canonical correspondence analysis (CCA) was performed by calling the “cca” function from the vegan package [44] via phyloseq. A total of six physico-chemical parameters (lignin content, pH, total C, K, P and C to N ratio) were chosen by the CCA model as those that significantly differentiated Mencía grape marc bacterial communities over the course of the vermicomposting process. The abovementioned physico-chemical parameters were determined as described in Gómez-Brandón et al. [24]. For the CCA model, the *P* values from the permutation tests were less than 0.05, indicating that the CCA model explained more variance of the bacterial communities during grape marc vermicomposting than expected by chance.

All analyses were performed in R version 3.5 [45], while all figures were created using the R package ggplot2 [46].

## 3. Results

### 3.1. Earthworm Biomass and Microbial Activity during Vermicomposting of Mencía Grape Marc

**Figure 1:**
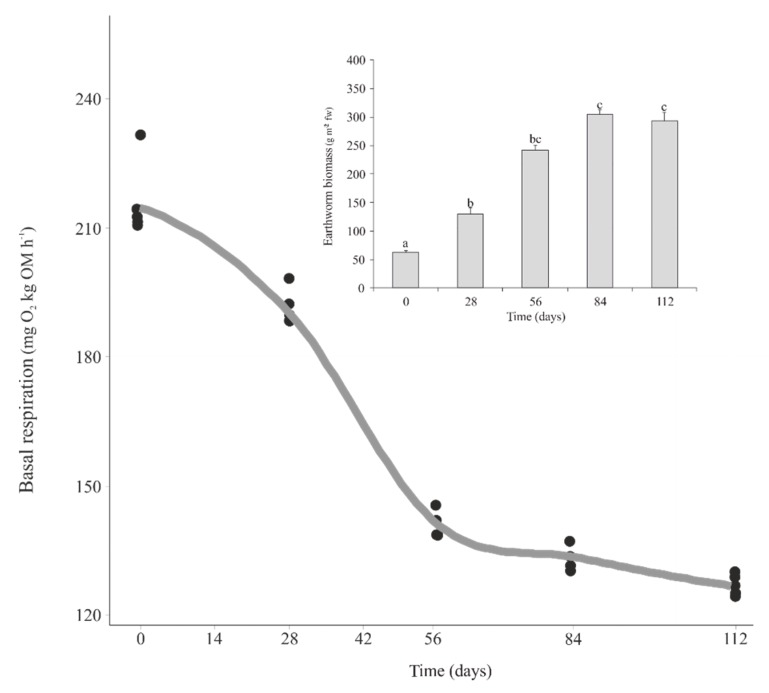
Changes in microbial activity measured as basal respiration during vermicomposting of grape marc derived from the red winemaking process of the grape variety Mencía. Individual values (*n* = 5) are plotted for each time point, and the curve was plotted using the “loess” smoothing method in ggplot2 [46]. The inset shows changes in earthworm biomass during the process. Earthworm biomass values are presented as means ± standard error (*n* = 5). Letters indicate significant differences between time points (Tukey HSD test).

There was a continuous and significant increase of the earthworm biomass from the beginning of the trial until day 84 (*p* < 0.05), after which the earthworm biomass did not change significantly (Figure 1 inset). Microbial activity assessed as basal respiration decreased during vermicomposting, with the greatest reduction between days 0 and 56 (Figure 1).

### 3.2. Changes in Bacterial Community Composition during Vermicomposting of Mencía Grape Marc

When comparing the bacterial community composition of the fresh grape marc (day 0) to that from day 14, we found that a total of 420 ASVs significantly differed in abundance (Figure S3, Table S1). A lower number of ASVs were identified as significantly different for the remaining time to time comparisons (354 ASVs for days 14–28, 396 ASVs for days 28–42, and 326 for days 42–91, Figure S3, Table S1).

**Figure 2:**
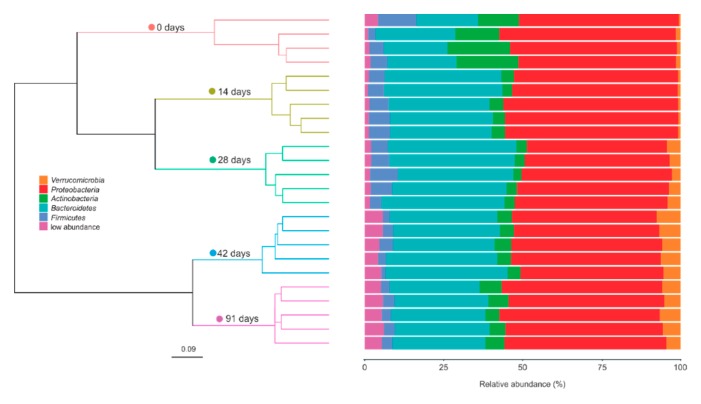
Changes in the bacterial community composition (phylum level) during vermicomposting of grape marc derived from the red winemaking process of the grape variety Mencía. The dendrogram represents the dissimilarity of bacterial communities at ASV levels (unweighted UniFrac distances, Ward method). Bars represent the relative abundance of dominant bacterial phyla. Bacterial phyla with lower relative abundances (<1%) were grouped together.

The bacterial community composition of the initial grape marc (day 0) was dominated by the phylum *Proteobacteria* (nearly 50% of the sequences, Figure 2). *Proteobacteria* continued to make up the most significant proportion of the bacterial communities on day 14 and, to a lesser extent, between days 28 and 91 (Figure 2, Table S2). During this timeframe, the differential abundances of the classes *Gamma*- and *Alphaproteobacteria* were notably reduced between days 28 and 42 and remained without significant changes until day 91 (Figure S4). In contrast, *Deltaproteobacteria* showed a higher differential abundance after 14 days of vermicomposting, and such levels were kept throughout the entire process (Figure S4, Table S3).

Besides *Proteobacteria*, ASVs from the phyla *Bacteroidetes* and *Actinobacteria*, with minor contributions of *Firmicutes* and *Verrucomicrobia*, accounted for about another half of the sequences of the fresh grape marc (Figure 2). *Bacteroidetes* slightly increased in abundance within the first 14 days of vermicomposting, before decreasing significantly between time periods 28–42 and 42–91 days (Table S2). A similar trend was observed for the class *Bacteroidia*, except for the fact that in this case no significant changes were recorded from day 42 until the end of the trial (Figure S4, Table S3). A significant decrease in the differential abundance of *Actinobacteria* was recorded after 14 days of vermicomposting and no more noticeable differences were recorded for the duration of the experiment (Table S2). There was a shift in the composition of *Actinobacteria* broken into the class level (Figure S4, Table S3). The differential abundances of the classes *Actinobacteria*, *Acidimicrobiia* and *Thermoleophilia* were sharply reduced after 14 days, and while the class *Actinobacteria* remained without noticeable changes until day 91, higher abundances for *Acidimicrobiia* and *Thermoleophilia* were recorded on days 42 and 91 compared to the middle time points (Figure S4, Table S3). *Firmicutes* showed similar values in terms of abundance until day 28, followed by a pronounced reduction between days 28 and 42 while no more changes were observed until the end of the trial (Table S2). Within *Firmicutes*, there was a significant increase in the abundance of classes *Clostridia* and *Bacilli* after 14 days of vermicomposting, followed by a reduction between days 14 and 28 in case of *Bacilli* whereas no changes were reported for *Clostridia* for this time period (Figure S4, Table S3). Later on, the abundance of both bacterial classes significantly decreased between days 28 and 42, and no more noticeable differences were reported for the duration of the trial (Figure S4, Table S3). In case of the class *Negativicutes*, its abundance significantly increased after 28 days of vermicomposting and no more significant changes were reported until the end of the experiment (Figure S4, Table S3). The same trend was observed for the phylum *Verrucomicrobia* (Table S2).

Other bacterial phyla that appeared in lower abundance than the abovementioned ones also varied significantly between time pairs during vermicomposting of Mencía grape marc (Tables S2 and S3). For instance, the phylum *Planctomycetes* (class *Planctomycetacia*) was greatly reduced after 14 days of vermicomposting, followed by a pronounced increase between days 28 and 42 and remaining without noticeable changes until day 91 (Figure S4, Tables S2 and S3). In case of the phylum *Acidobacteria* (class *Acidobacteriia*), there was a significant increase after 14 days and no more changes were reported for the duration of the trial (Figure S4, Tables S2 and S3). However, a significant increase in the abundance of the phylum *Armatimonadetes* (class *Fimbriimonadia*) was observed later on, between 14–28 days and 28–42 days (Figure S4, Tables S2 and S3).

### 3.3. Changes in α- and β-Diversity during Vermicomposting of Mencía Grape Marc

**Figure 3:**
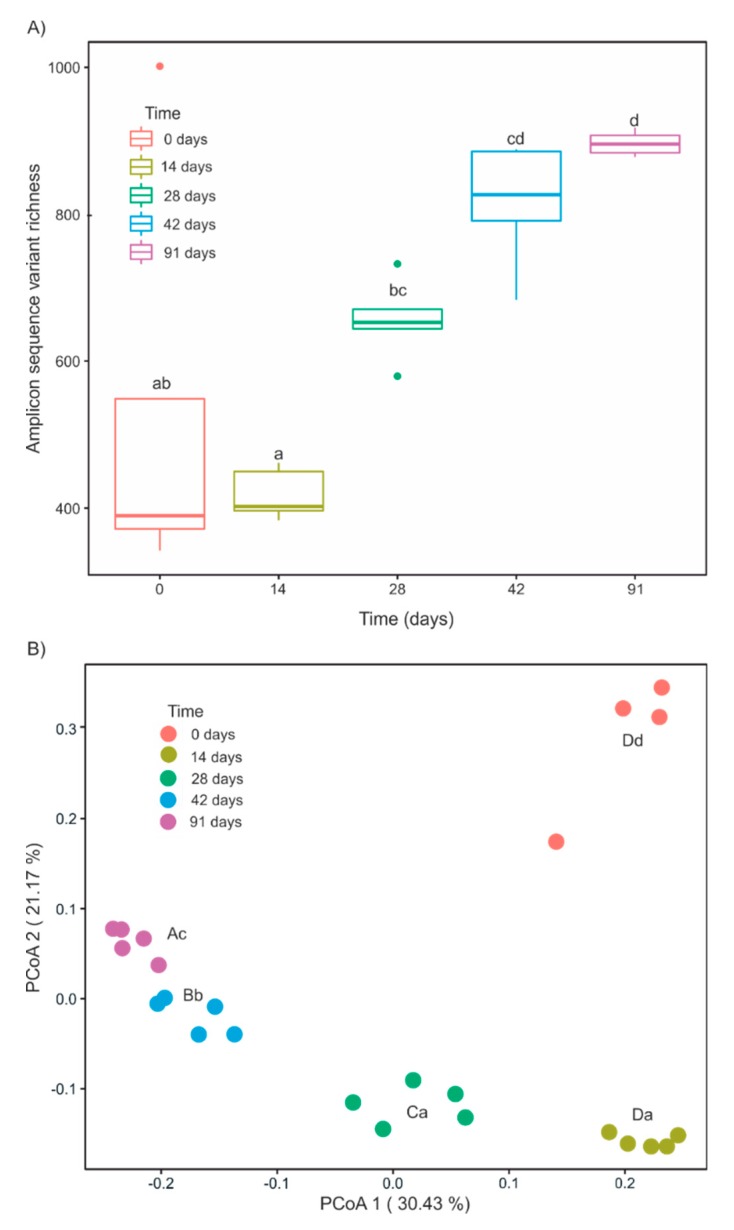
Changes in bacterial α-diversity and β-diversity during vermicomposting of grape marc derived from the red winemaking process of the grape variety Mencía. (**A**) α-diversity is described in terms of amplicon sequence variant (ASV) richness. Letters indicate significant differences between time points (Tukey HSD test). (**B**) β -diversity is shown with principle coordinate analysis of weighted UniFrac distances. Capital and lowercase letters indicate significant differences between the time points in PCoA1 and PCoA2 scores respectively (Tukey HSD test, FDR corrected).

Although α-diversity did not increase significantly between days 0 and 14, steady and significant increases were observed between days 14 and 91 in ASV richness (Figure 3A), Chao1 richness, Shannon diversity, and Faith phylogenetic diversity (Figure S5, Table 1). These increases in α-diversity were also reflected in different patterns in phylogenetic and taxonomic β-diversity (Figure 3B and Figure S5).

Principle coordinate analysis showed that bacterial community composition from the fresh grape marc (day 0) differed significantly from that of vermicomposted grape marc (days 14–91) along the second dimension that accounted for 21.17% of the total variance (Figure 3B, Table 1). The first dimension that explained 30.43% of the total variance reflected the changes in bacterial community composition between the different stages of the vermicomposting process (Figure 3B). Significant changes in bacterial community composition were found between the earlier and middle time points (14 and 28 days, respectively) of the active phase of vermicomposting (Figure 3B). The 14- and 28-day samples also grouped separately from the 42- and 91-day vermicomposts (i.e., maturation stage) which clustered quite closely together in the negative side of the first dimension (Figure 3B). These trends also held true using unweighted UniFrac, Bray-Curtis, and Jaccard distance matrices (Figure S5, Table 1).

### 3.4. Core Microbiome during Vermicomposting of Mencía Grape Marc

**Figure 4:**
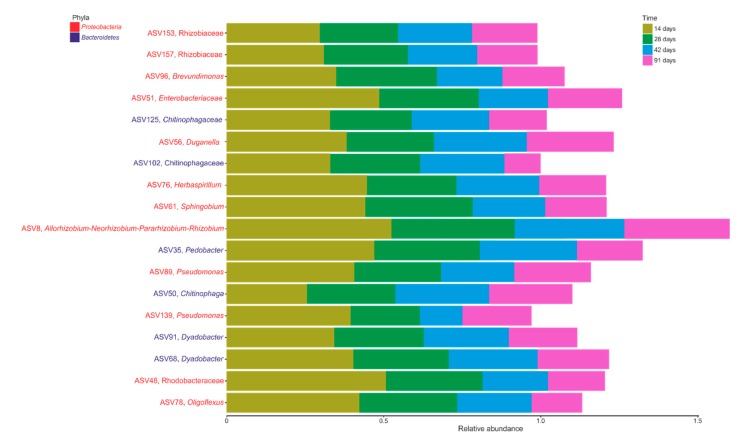
Relative abundance (%) of ASVs (phylum and genus or most inclusive taxonomy found) from the core microbiome of vermicomposting of grape marc derived from the red winemaking process of the grape variety Mencía across days 14, 28, 42, and 91.

A total of eighteen ASVs were identified as the bacterial core microbiome during vermicomposting of Mencía grape marc, and they were present in all of the samples from days 14, 42, 28, and 91 (Figure 4). These ASVs represented 1.61% of total ASVs and 16.25% of all sequences. The initial grape marc (day 0) was not considered within the core microbiome because this sample was not subjected to the action of earthworms. Twelve of these ASVs belonged to the phylum *Proteobacteria* and the other six to the phylum *Bacteroidetes* (Figure 4). The phylum *Proteobacteria* comprised ASVs from the families *Rhizobiaceae* (ASV153 and ASV157), *Enterobacteriaceae* (ASV51) and *Rhodobacteraceae* (ASV48), and from the genera *Brevundimonas* (ASV96), *Duganella* (ASV56), *Sphingobium* (ASV61), *Allorhizobium-Neorhizobium-Pararhizobium-Rhizobium* (ASV8), *Pseudomonas* (ASV89 and ASV139) and *Oligoflexus* (ASV78). The abundance of the ASVs 48, 51, 61, 96, and 78 was significantly different between days 28 and 42 (Table S1). Moreover, the ASVs 78 and 139 differed in abundance between days 42 and 91 (Table S1).

Among the *Bacteroidetes* present, two ASVs belonged to the family *Chitinophagaceae* (ASV125 and ASV102), and the remaining ASVs to the genera *Pedobacter* (ASV35), *Chitinophaga* (ASV50) and *Dyadobacter* (ASV91 and ASV68). The abundance of the ASVs 50 and 102 varied significantly between days 14 and 28 (Table S1). Significant differences in the abundance of the ASVs 35 and 102 were also found between days 42 and 91 (Table S1).

### 3.5. Functional Profiles during Vermicomposting of Mencía Grape Marc

**Figure 5:**
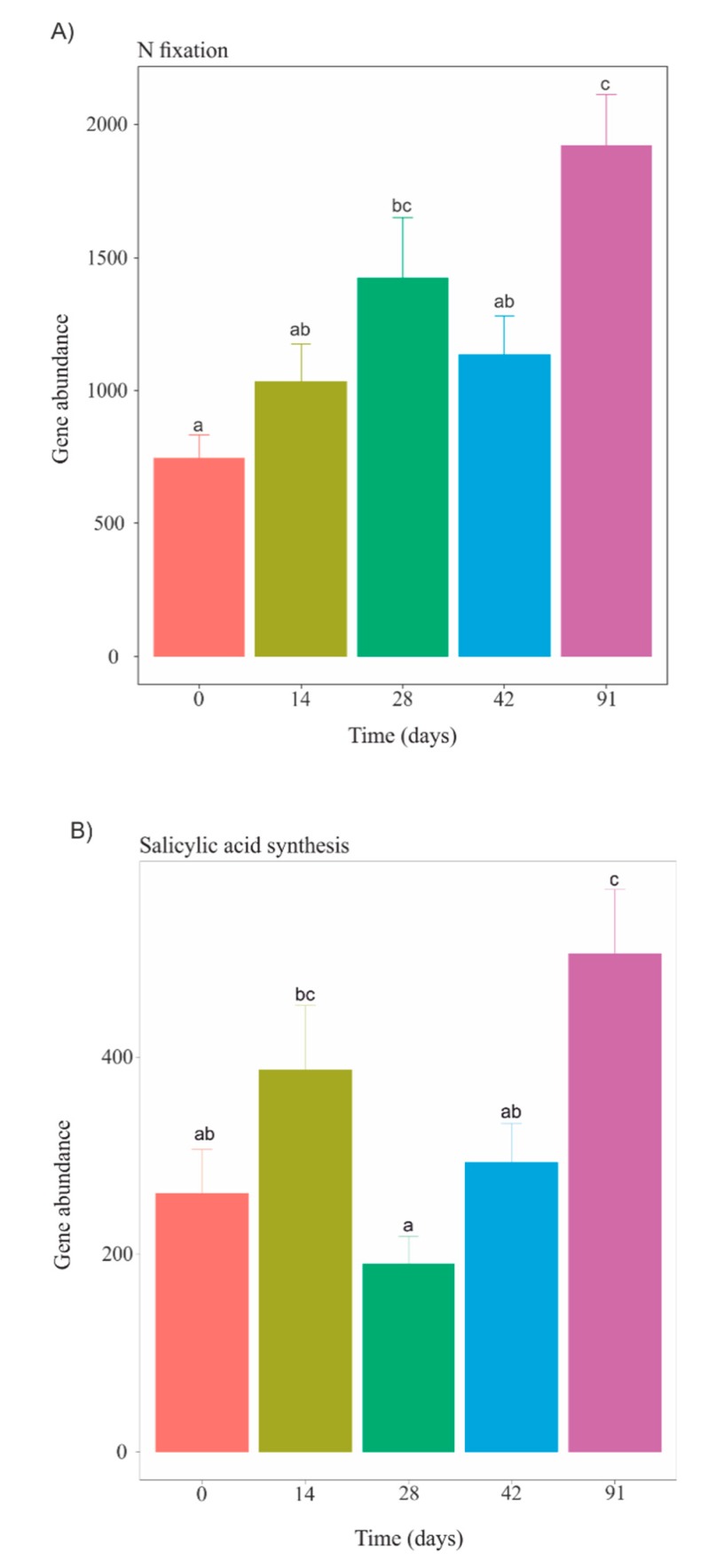
Changes in gene abundance of PICRUSt-predicted enzyme-level genes involved in nitrogen fixation (**A**) and synthesis of salicylic acid (**B**) during vermicomposting of grape marc derived from the red winemaking process of the grape variety Mencía. Letters indicate significant differences between time points (Tukey HSD test).

Metagenomic predictions using PICRUSt showed distinct functional profiles over the course of vermicomposting of Mencía grape marc with regard to the abundance of specific functional genes like those involved in nitrogen fixation and synthesis of salicylic acid (Figure 5). Overall, the relative abundance of genes related to nitrogen fixation increased throughout the vermicomposting process reaching the highest level on day 91 (Figure 5A). Genes involved in the synthesis of salicylic acid were significantly reduced on day 28 compared to day 14, followed by a significant increase on day 91 (Figure 5B).

### 3.6. Influence of Physico-Chemical Variables on Bacterial Community Composition during Vermicomposting of Mencía Grape Marc

**Figure 6:**
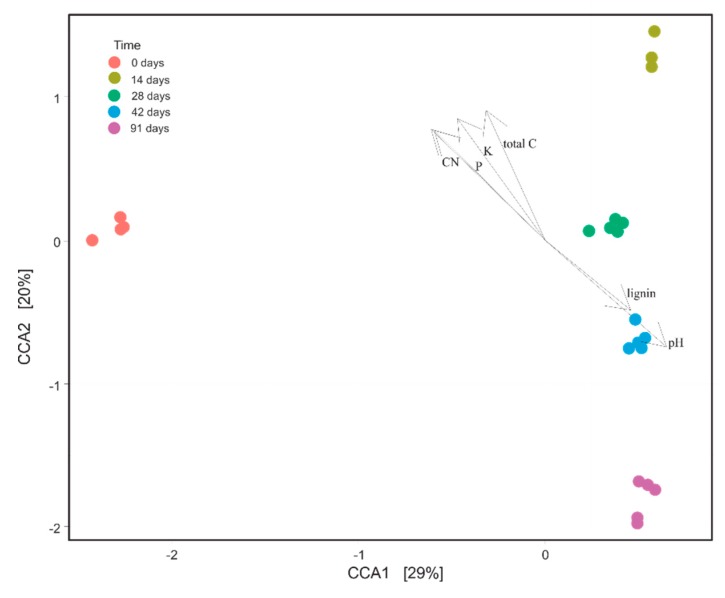
Canonical correspondence analysis showing the selected physico-chemical parameters (represented as vectors) shaping the bacterial community composition during vermicomposting of grape marc derived from the red winemaking process of the grape variety Mencía. Based on permutation tests, all the physico-chemical variables retained in the model were significant (*p* < 0.05) in constraining bacterial communities.

The CCA model accounted for 49% of the total variation and based on permutation tests, six explanatory physico-chemical variables were retained in the model as significant in shaping bacterial community composition during vermicomposting of Mencía grape marc (Figure 6). The first axis clearly discriminated between the initial grape marc (t = 0, negative side) and the vermicomposted grape marc subjected to the action of earthworms at the different sampling times (days 14–91, Figure 6). pH was positively and highly associated with this axis (R^2^ = 0.652), as indicated by the length and direction of its vector (Figure 6). The second axis clearly differentiated between the active (i.e., 14 and 28 days) and the maturation (i.e., 42 and 91 days) stages of vermicomposting, which fell in the positive and negative sides of this dimension respectively (Figure 6). Total C (R^2^ = 0.904) and K (R^2^ = 0.850) followed by P (R^2^ = 0.771) were positively and highly related to the second axis, while pH (R^2^ = −0.743) was negatively associated to this axis (Figure 6).

## 4. Discussion

In the present study, we provide a detailed insight into the vermicomposting of grape marc derived from the red winemaking process of Mencía grapes from a microbial-molecular perspective. With the use of a time-series sampling and high-throughput sequencing, we dig deeper into the dynamics and functional capacities of the bacterial communities that orchestrate the vermicomposting process of this winery by-product.

The composition of bacterial communities in the Mencía grape marc displayed distinct temporal variations during the vermicomposting process at ASV, phylum and class levels. We observed that the bacterial communities involved in the process changed quickly within the first 14 days of vermicomposting since a higher number of ASVs differed in abundance between the initial grape marc (day 0) and day 14 (Figure S3, Table S1), when compared to the other pairs of time points (days 14–28, 28–42, and 42–91). Accordingly, the fresh grape marc that has not been affected by the earthworms clustered separately from days 14 and 28 (Figure 2 and Figure 6) that reflect the active phase of the vermicomposting process. On the other end, the bacterial community composition of the final group, days 42 and 91, was relatively similar (Figure 2) and likely reflected bacteria associated with the aging process of the casts that take place during the maturation phase [9]. pH was found to be one of the major driving factors discriminating the composition of bacterial communities from the fresh (day 0) and the vermicomposted (days 14–91) grape marc (Figure 6), as well as between the active (days 14 and 28) and the maturation (days 42 and 91) stages of vermicomposting (Figure 6). This underscores the key role of pH in the dynamics of the vermicomposting process probably due to the fact that it is often correlated with underlying environmental factors influencing the microbial community such as nutrient availability, and/or the synthesis and activity of enzymes [47,48].

We observed that *Proteobacteria* was the most prevalent phylum after 14 days and throughout the entire process (Figure 2), as previously shown by Lv et al. [15] for vermicomposting of sewage sludge and cattle dung and Gopal et al. [12] for vermicomposting of lignin-rich coconut leaves. At the earlier time point of 14 days, the community composition mainly reflects bacteria that have recently passed through the intestines of earthworms and been excreted. These egested materials rapidly decompose and constitute a source of both nutrients and microorganisms, which may thus affect the rate of decomposition within the vermicomposting system [3,4]. It is then expected that during the active phase labile nutrient pools released from the egested casts support the growth of copiotrophic bacteria characterized by faster rates of carbon turnover and specialized on rich and soluble substrates that, eventually, will be replaced by oligotrophic bacteria with a higher substrate utilization efficiency and able to metabolize the remaining recalcitrant substrates in the casts during the maturation stage. In agreement with this, copiotrohic bacteria, like those belonging to the classes γ-*Proteobacteria* and *Bacteroidia* [49], showed higher abundances during the active phase of vermicomposting of grape marc (days 0 to 28, Figure S4). However, bacteria associated with oligotrophic environments such as those from the classes *Acidimicrobiia*, *Planctomycetacia* and *Fimbriimonadia* [49] were more abundant in the later stages of the process (days 42 to 91, Figure S4). Indeed, evidence has emerged of bacteria with lignin-decomposing abilities, albeit to a lesser extent than fungi, from members belonging to phyla *Acidobacteria* and *Planctomycetes* [50]. These data taken together underscore how quickly vermicomposting affects the bacterial communities from the initial substrate and provide a strong example of microbial succession driven by changes in the organic carbon source during vermicomposting of grape marc derived from red winemaking processes.

In addition to these rapid bacterial composition changes, there was also an increase in bacterial diversity, both taxonomic and phylogenetic, from day 14 until the end of the experiment. This is in agreement with previous studies in which vermicompost had higher bacterial diversity than the initial feedstock and/or composted materials [12,13,15,17]. Furthermore, our results match with previous findings from our research group dealing with vermicomposting of grape marc derived from white winemaking of Albariño grapes [27]. In both cases, the diversity of the starting material (white and red grape marc) is lower than in other frequently composted solid wastes such as manure or sewage, but the Mencía grape marc used in the present study showed a 5-fold higher taxonomic diversity compared to Albariño white grape marc. Such differences in terms of microbial diversity are probably due to the different procedures followed during the winemaking process of red and white grape varieties. During red wine vinification, skins and seeds remain in contact with the fermentation broth for several days, whereas during white winemaking, the fermentation of the grape juice occurs with minimal or no contact with the grape marc. This prolonged contact with the fermentation broth in red winemaking likely explains the increased microbial diversity in the resulting grape marc.

Unravelling the compositional core of a microbial consortium is the first step in defining a “healthy” community and may help to predict community responses to perturbation [51], which it is essential in the context of our study considering the potential use of grape marc vermicompost as soil organic amendment and/or plant growth promoter. Within the core microbiome of Mencía grape marc (days 14, 28, 42, and 91), we found members of the phylum *Proteobacteria* are known to be capable of nitrogen-fixation. Members of the family *Enterobacteriaceae* and certain strains from genera *Pseudomonas* and *Duganella* have the potential of associative nitrogen fixation [52]. Several strains of *Pseudomonas* are known to confer plant-disease suppression [53]. The family *Rhizobiaceae* and the genera *Allorhizobium-Neorhizobium-Pararhizobium-Rhizobium*, well-known for nitrogen fixation through the formation of nodules in the host plant’s root system [52], along with members of the phylum *Bacteroidetes* like the genera *Pedobacter* and *Dyadobacter* were additionally present in the core microbiome of grape marc. Interestingly, there is evidence to suggest that strains of these two latter bacterial genera have antagonistic activities against phytopathogens and could provide plant protection [54,55], even though the mechanism for this suppression is still unclear. Accordingly, analysis with PICRUSt showed increases in specific metabolic processes potentially beneficial for plant growth and development, including nitrogen fixation and the synthesis of salicylic acid, at the end of the vermicomposting process of Mencía grape marc (91 days, Figure 5). Indeed, salicylic acid has long been known to reduce plant stress by promoting the activation and the modulation of plant defense responses and increasing the antioxidant activity of plants [56].

These variations in functional diversity of the bacterial communities over the course of vermicomposting could provide a plausible microbial-derived mechanism by which improved plant performance occurs when grown in vermicompost [12,57,58,59]. In line with this, Song et al. [58] found that adding vermicompost enhanced the beneficial effects of plant growth-promoting rhizobacteria on both soil and crop, but the extent of this promotion varied with the dose of vermicompost and the crop type. As such, it should be noted that the metabolic functions of vermicompost microbiomes may also be different depending on the initial substrate, earthworm species and/or vermicomposting method, which can have ultimate consequences on the benefits of vermicompost when used as a soil amendment and/or plant growth promoter.

## 5. Conclusions

In conclusion, vermicomposting of grape marc obtained from the red winemaking process of Mencía grapes resulted in a stable but richer and more diverse bacterial community, which supports the use of grape marc vermicompost for sustainable practices in the wine industry by disposing of this high-volume winery by-product and capturing its value to improve soil fertility.

## Figures and Tables

**Table 1:** Results from mixed-effects models are shown for α- and β-diversity indices. Significance was determined using ANOVA. For each test, we report the relevant F statistic (*F_4,19_*) and significance (*p*(>*F*)). Degrees of freedom were constant across all tests (numerator degrees of freedom: 4, denominator degrees of freedom: 19).

Alpha Diversity		F_4,19_	*p*(>F)
	Observed	10.45	0.0003
	Chao1	9.11	<0.0001
	Shannon	39.50	<0.0001
	Faith PD	13.34	0.0001
Beta diversity		F_4,19_	*p*-value
Unifrac–unweighted	PCoA1	203.58	<0.0001
	PCoA2	114.67	<0.0001
Unifrac-weighted	PCoA1	175.00	<0.0001
	PCoA2	71.91	<0.0001
Bray-Curtis	PCoA1	158.46	<0.0001
	PCoA2	339.88	<0.0001
Jaccard	PCoA1	254.34	<0.0001
	PCoA2	146.48	<0.0001

## Supplementary Materials

The following are available online at https://www.mdpi.com/2076-2607/7/10/473/s1.
