# Supplementary material for: Rapid Bacterial Community Changes during Vermicomposting of Grape Marc Derived from Red Winemaking
Source: Microorganisms. 2019 Oct 19;7(10):473. doi: 10.3390/microorganisms7100473 (PMC6843324; doi:10.3390/microorganisms7100473)
Supplement: Supplementary file 1 [file microorganisms-07-00473-s001.zip › Supplementary Figures.pdf]

Supplementary Figures

Rapid bacterial community changes during vermicomposting of grape marc derived from red winemaking

María Gómez Brandón[1,*, †], Manuel Aira[1, †], Allison R. Kolbe[2], Nariane de Andrade[3], Marcos Pérez-Losada[2,4,5], Jorge Domínguez[1]

[1]Grupo de Ecoloxía Animal (GEA), Universidade de Vigo, E-36310, Spain.

[2]Computational Biology Institute, Milken Institute School of Public Health, George Washington University, Ashburn, VA 20147, USA.

[3]Departamento de Solos, Universidade Federal de Santa María, Río Grande do Sul, 97105-900, Brasil

[4]CIBIO-InBIO, Centro de Investigação em Biodiversidade e Recursos Genéticos, Universidade do Porto, Campus Agrário de Vairão, 4485-661 Vairão, Portugal.

[5]Department of Biostatistics and Bioinformatics, Milken Institute School of Public Health, George Washington University, Washington, DC 20052, USA

*Corresponding author: mariagomez@uvigo.es

†The first two authors contributed equally to this paper.

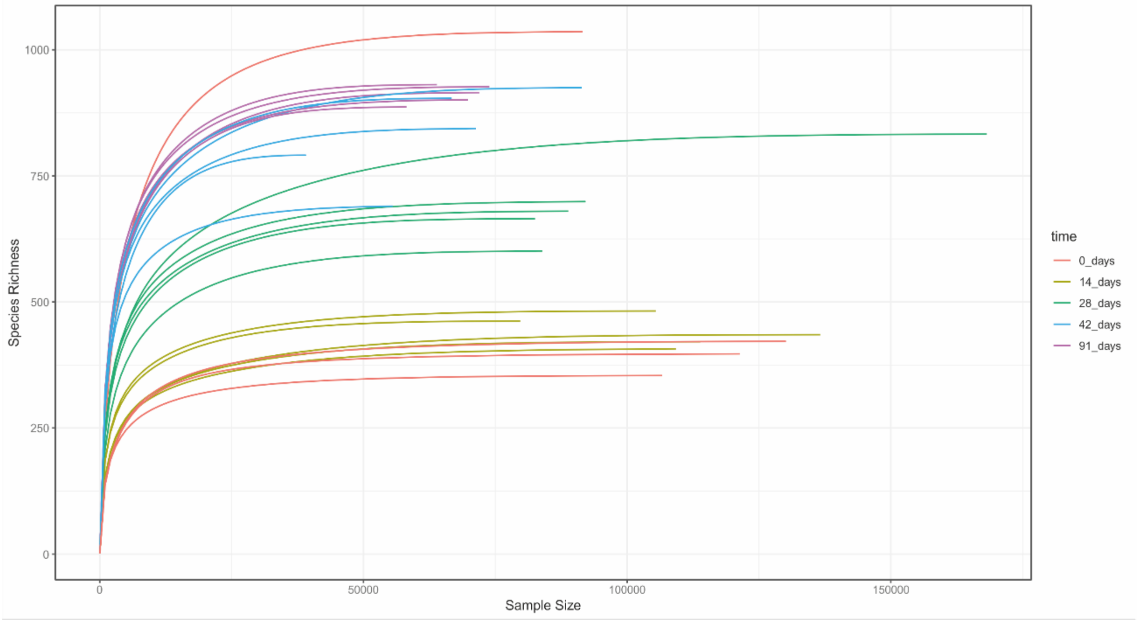

Figure S1 Rarefaction curves showing the number of amplicon sequence variants (ASVs) found in each sample during vermicomposting of grape marc derived from the red winemaking process of the grape variety Mencía. These curves indicate that the sequencing depth was optimal for all of the samples in the full data set.

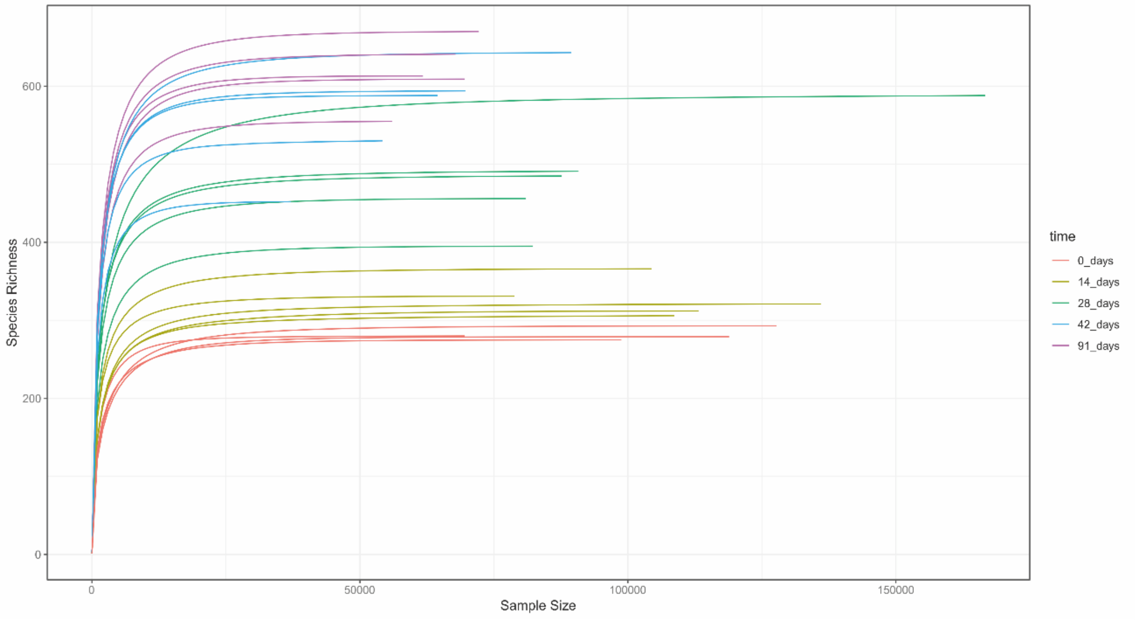

Fig. S2 Rarefaction curves showing the number of amplicon sequence variants (ASVs) found in each sample during vermicomposting of grape marc derived from the red winemaking process of the grape variety Mencía. These curves indicate that the sequencing depth was optimal for all of the samples in the filtered data set.

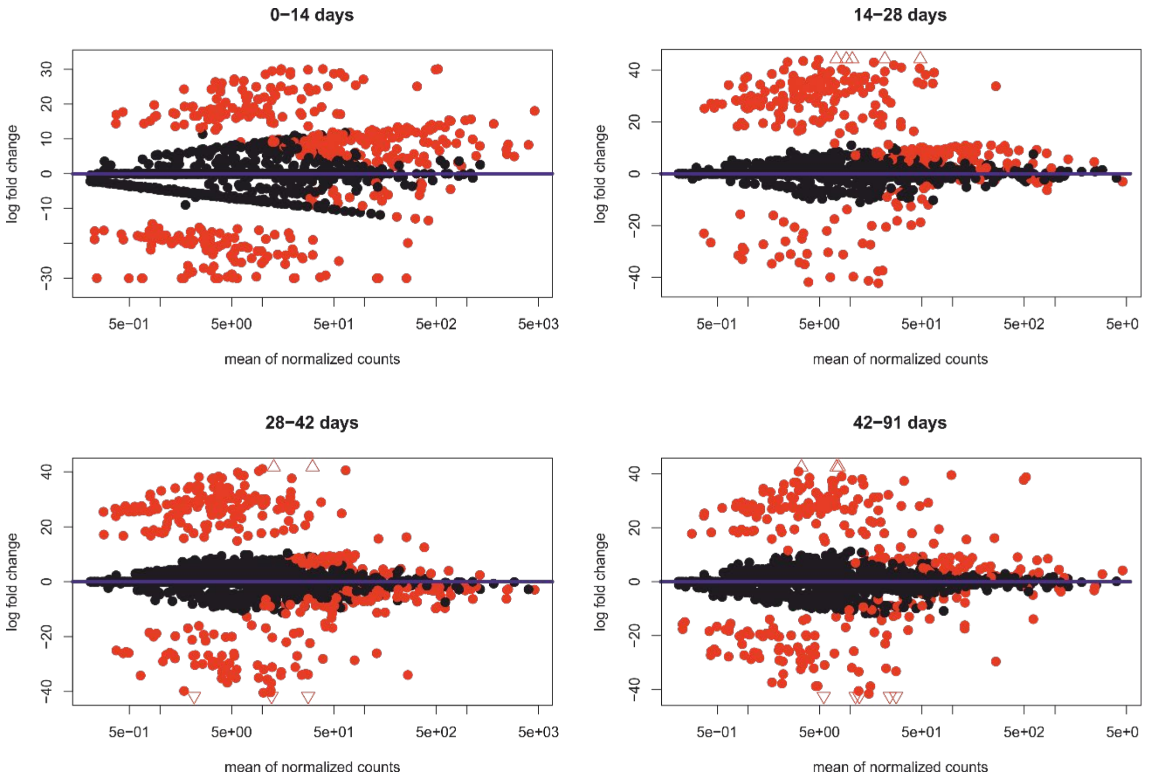

Fig. S3 MA-plots of the estimated fold change in ASVs over average expression strength during vermicomposting of grape marc derived from the red winemaking process of the grape variety Mencía. Small triangles at the top and bottom of the plots indicate points that would fall outside of the plotting window.

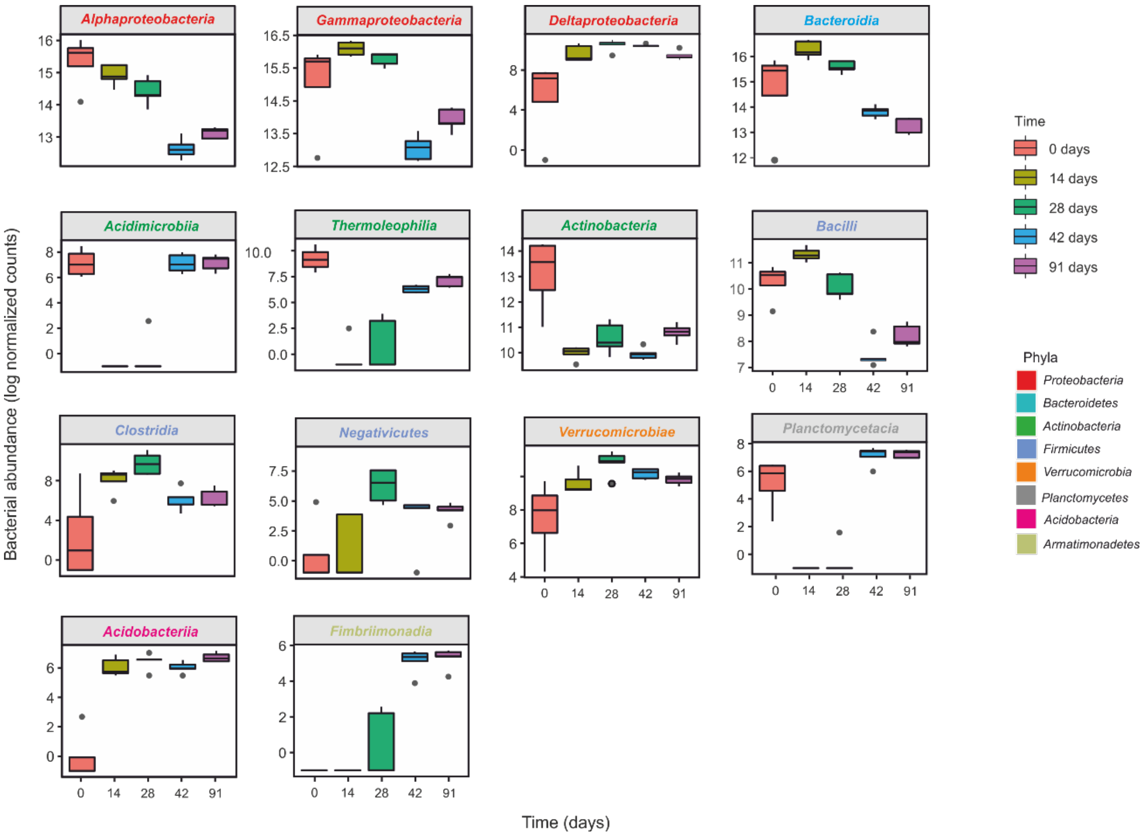

Fig. S4 Boxplots showing the changes in the normalized abundance of bacterial classes during vermicomposting of grape marc derived from the red winemaking process of the grape variety Mencía. Abundance changes are expressed as log_2fold. A specific colour is given to the name of the different bacterial classes according to the phylum they belong to.

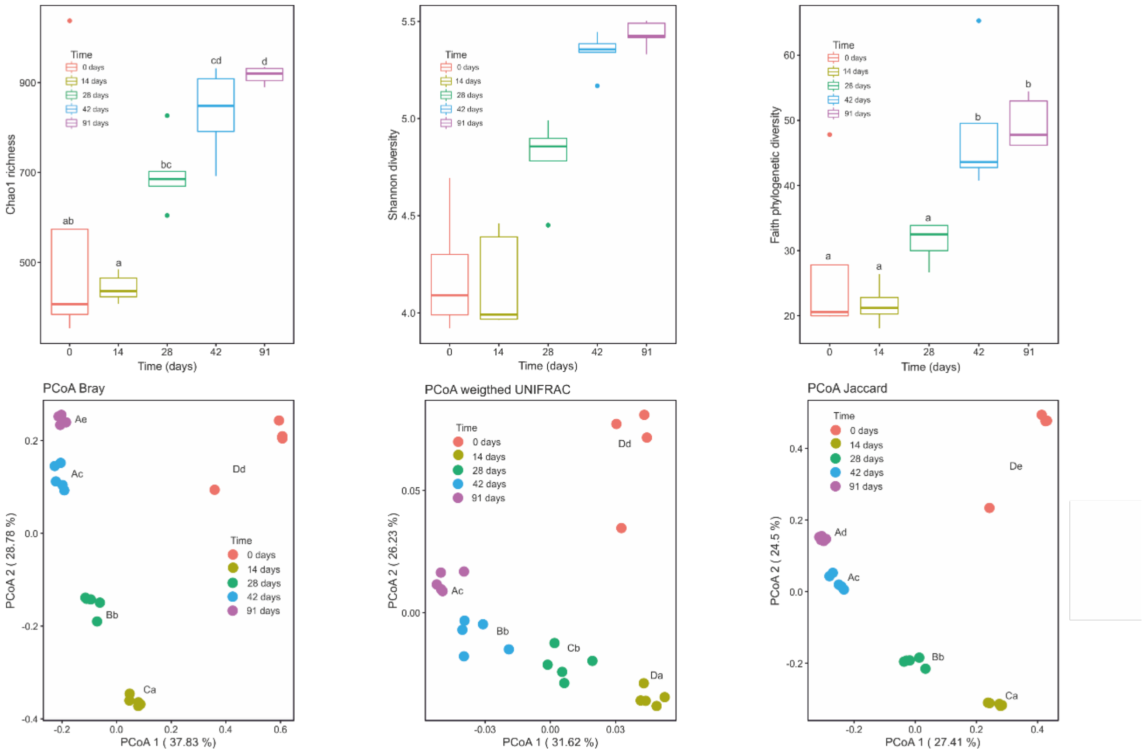

Fig. S5 Additional estimates of bacterial α-diversity and β-diversity during vermicomposting of grape marc derived from the red winemaking process of the grape variety Mencía. α-diversity increased over time regardless of estimation method, including Chao1 richness (A), Shannon diversity (B), and Faith phylogenetic diversity (C). Letters in (a)-(c) denote significant differences between the different stages of the vermicomposting process (Tukey HSD test). Principle coordinate analysis with Bray-Curtis (D), weighted UniFrac (E), and Jaccard (F) showed significant differences between stages of vermicomposting. Different capital and lowercase letters indicate significant differences between the time points in PCoA1 and PCoA2 scores, respectively.
